# The Effect of PPARγ rs1801282 Variant on Mortality Risk Among Asians With Chronic Kidney Disease: A Cohort Study and Meta-Analysis

**DOI:** 10.3389/fgene.2022.705272

**Published:** 2022-02-21

**Authors:** Wei-Teing Chen, Chih-Chien Chiu, Dung-Jang Tsai, Pi-Shao Ko, Meng-Chang Lee, Hsiao-Ting Lin, Ying-Kai Chen, Wen Su, Yuh-Feng Lin, Sui-Lung Su

**Affiliations:** ^1^ Division of Chest Medicine, Department of Medicine, Cheng Hsin General Hospital, Taipei, Taiwan; ^2^ National Defense Medical Center, Department of Medicine, Tri-Service General Hospital, Taipei, Taiwan; ^3^ Division of Infectious Diseases, National Defense Medical Center, Department of Internal Medicine, Taoyuan Armed Forces General Hospital, Taoyuan, Taiwan; ^4^ School of Public Health, National Defense Medical Center, Taipei, Taiwan; ^5^ National Defense Medical Center, Graduate Institute of Life Sciences, Taipei, Taiwan; ^6^ Division of Nephrology, Department of Medicine, Zuoying Branch of Kaohsiung Armed Forces General Hospital, Kaohsiung, Taiwan; ^7^ National Defense Medical Center, Graduate Institute of Aerospace and Undersea Medicine, Taipei, Taiwan; ^8^ Division on Nephrology, Department of Internal Medicine, School of Medicine, College of Medicine, Taipei Medical University, Taipei, Taiwan; ^9^ Division of Nephrology, National Defense Medical Center, Department of Medicine, Tri-Service General Hospital, Taipei, Taiwan

**Keywords:** chronic kidney disease, mortality, peroxisome proliferator-activated receptor gamma, gene polymorphism, meta-analysis, trial sequential analysis

## Abstract

**Background:** Chronic kidney disease (CKD) is a public health issue, and an independent risk factor for cardiovascular disease. The peroxisome proliferator-activated receptor gamma (PPARG) plays an important role in the cardiovascular system. Previous studies have examined one important exon polymorphism, Pro12Ala, in PPARG with respect to mortality of CKD patients, but the results were inconsistent and current evidence is insufficient to support a strong conclusion. This study aimed to examine the correlation between Pro12Ala gene polymorphism and mortality among Asians with CKD by trial sequential analysis (TSA).

**Methods:** The research was divided into observational research and meta-analysis. For the cohort study, 767 subjects from dialysis centers in Taipei were selected as samples, and tracked from December 2015 to February 2017. For the meta-analysis, relevant literature from “PubMed” and “Embase” databases (until December 2016), was searched and TSA was used to verify the results. In order to achieve the best evidence hierarchies, our retrospective cohort study was added to the meta-analysis and the TSA.

**Results:** The combined sample size for Asian was 1,685 after adding our cohort study, and there was no significant correlation between PPARG Pro12Ala and mortality by the allele model (RR: 0.85, 95% CI: 0.39–1.83, I^2^ = 79.3%). Under the parameter setting with the RR value of 1.5, TSA estimation presented that the cumulative sample size entered into the futility area, and it confirmed the conclusion in this study.

**Conclusion:** We found that PPARG Pro12Ala gene polymorphism was not related to mortality in CKD Asians patients, and validated our conclusion using TSA after adding our sample.

## 1 Introduction

Chronic kidney disease (CKD) is a public health issue around the globe with a prevalence of approximately 10% (Coresh et al., 2007). Compared with the general population, patients with CKD have a higher risk of cardiovascular diseases and mortality (Imai et al., 2009). The probability of death in patients with CKD is 8–10 times higher than the general population and increases with renal dysfunction (Zhang et al., 2012a). Based on prior evidence, genetic factors such as ethnicity and familial inheritance play vital roles in patients with deterioration of CKD (Imperatore et al., 2000; Langefeld et al., 2004; Parikh et al., 2008; MacCluer et al., 2010; Tsai et al., 2010; Jha et al., 2012). Therefore, it is critical to assess CKD-related gene polymorphisms in reducing the risk of death.

PPARG is a transcription factor the main functions of which include regulation of differentiation and proliferation of fat cells and fatty acids, and carbohydrate metabolism (Ahmadian et al., 2013). PPARG can reduce inflammatory factor production, increase glucose utilization rate in muscles, decrease gluconeogenesis in the liver, and increase insulin sensitivity (Ruan et al., 2008). PPAR gamma activators also act as ordinary hypoglycemic agents (such as Thiazolidinedione), as they promote the secretion of adiponectin and adipsin by adipocytes, and inhibit inflammatory responses at the same time resulting in the rise of insulin sensitivity of muscle tissues (Corzo and Griffin, 2013). In addition to diabetes treatment, PPARG activation can also decrease renal fibrosis and inflammation, reduce salt and water retention in the renal tubules to prevent swelling of the stromal cells and reduce damage to kidney cells (Yki-Järvinen, 2004). PPARG dysfunction may lead to a decline in kidney function, so PPARG Pro12Ala polymorphism may be associated with a risk of death in patients with CKD.

From the above, we can infer that the leading cause of death in patients with CKD is cardiovascular disease, accounting for 53.1% of the all-cause mortality rate (Go et al., 2004). The correlation between PPARG Pro12Ala (rs1801282C > G) and risk of death in patients with CKD has already been established in the literature (Yao et al., 2005; Szeto et al., 2008). However, the results of these studies are still conflicting. For example, Szeto et al. found that patients with C/C genotype survived better than those with other genotypes (Szeto et al., 2008), but Yao et al. found that patients with G allele had better survival (Yao et al., 2005). To shed light on this discrepancy, we performed a large-scale cohort study and meta-analysis to investigate the association between Pro12Ala gene polymorphism and mortality. The trial sequential analysis (TSA) was used to confirm the accuracy of the result. However, the trial TSA estimated that cumulative sample size (*n* = 918) did not reach the target sample size. In order to increase the sample size, our cohort study subjects (*n* = 767) were added to the meta-analysis.

## 2 Materials and Methods

### 2.1 Retrospective Cohort Study

#### 2.1.1 Ethical Issues

We initiated a population-based study at the Tri-Service General Hospital (TSGH), a medical teaching hospital of the National Defense Medical Centre in Taipei, Taiwan. The project was reviewed and approved by the institutional ethical committee of the Tri-Service General Hospital (TSGH-1-104-05-006). All subjects enrolled in the study provided written informed consent. It was not appropriate or possible to involve patients or the public in the design, or conduct, or reporting, or dissemination of our research.

#### 2.1.2 Subjects

The subjects were recruited from dialysis centers or clinics in Taiwan. All cases were undergoing dialysis and were diagnosed by professional physicians in 2015.

The exclusion criteria were as follows: 1) a dialysis period less of than 3 months; 2) cancer diagnosis; and 3) insufficient blood samples. Finally, a total of 767 samples were included in the study analysis.

Demographic data included the dialysis clinic, age, gender, education level, diabetes, history of hypertension, duration of dialysis, and blood biochemical values (fasting glucose, triglycerides, cholesterol, leukocytes, erythrocytes, hemoglobin, urea nitrogen before dialysis, urea nitrogen after dialysis, creatine, albumin, urine protein, sodium, potassium, calcium, phosphorous, and eGFR) were collected from questionnaire and medical records. Death results were collected from the death registration files.

#### 2.1.3 Genomic DNA Extraction and Genotyping

Medical technologists or nurses collected 5 ml of intravenous blood samples from subjects. Genomic DNA was isolated using standard procedures for proteinase K (Invitrogen, Carlsbad, CA, United States) digestion and phenol/chloroform extraction from peripheral blood samples (Tan and Yiap, 2009).

Subjects were genotyped using the iPLEX Gold SNP assay to identify PPARG Pro12Ala polymorphisms (C/C, C/G, and G/G) (Perkel, 2008). We used inter-replication validation and intra-replication validation to assess the genotyping experiment quality. Inter-replication validation was repeated for 78 samples (approximately 5%), the concordance rate was 100%. After randomly selecting 10 samples, intra-replication validation was carried out using polymerase chain reaction according to a previously described protocol (Yang et al., 2015). Genotyping was carried out for a second time and the results of the first and second genotyping were compared. The concordance rate between the two genotyping methods was 100%.

#### 2.1.4 Statistical Analysis

Continuous variables were evaluated using Student’s t test and reported as means ± SDs. Genotypes and allelic frequencies were compared between patients with dialysis and healthy controls using the χ^2^ test or Fisher’s exact test where appropriate. The Cox proportional hazard model was used to estimate the HR and 95% CI for the risk of mortality. Allele type, genotype and dominant/recessive model were used to calculate the risk between genetic polymorphism and mortality. This study considered a *p* value of less than 0.05 as significant for all analyses. Statistical analyses were executed using R software, version 3.3.1 (R Project for Statistical Computing, Vienna, Austria).

The start and end points of the study were 31 December 2015 and 15 February 2017, respectively, and the length of the follow-up period was 14.5 months. Patients who dropped out of the study or transferred to another hospital during the study period were excluded. If the patient died during the study, the follow-up period was from 31 December 2015 to the time of death, and the result was recorded as “death”. If the patient was alive at the end of the study (15 February 2017), the follow-up period was from 31 December 2015 to 15 February 2017, and the result was recorded as “survived.”

#### 2.1.5 Meta-Analysis

##### Search Methods and Criteria for Study Consideration

The PRISMA checklist and the Meta-analysis on the Genetic Association Studies Checklist are described in Supplementary Table S1 (Moher et al., 2009). We evaluated the correlation between individuals carrying the major (C) and the minor (G) alleles of PPARG Pro12Ala and the risk of mortality in patients with CKD. In this study, we used the synonyms “PPARG Pro12Ala” and “chronic kidney disease” to search for PubMed and Embase for papers published up until 30 June 2021 (see Supplementary Table S2). The language of the articles was limited to English. In addition, we also manually scanned the reference lists of identified trials and review articles to identify additional candidate studies.

The criteria for study inclusion were as follows: 1) a cohort study; 2) samples presented a clear diagnosis, such as proteinuria, hypercreatinemia, low glomerular filtration rate and other abnormalities in renal function markers, or renal parenchymal lesion, computed tomography, ultrasonography, and other examination findings; 3) all-cause mortality records had to have a clear source; 4) the study contained the genetic distribution of target loci; 5) the Study population age was greater than 18 years; 6) papers without complete genetic information were removed.

##### Data Extraction

In the present study, two reviewers (Wei-Teing Chen and Dung-Jang Tsai) were responsible for the independent extraction of literature data. The data extracted included the last name of the first author, the year of publication, country, ethnicity of the study population, and gene frequency of surviving and deceased patients.

##### Statistical Analysis

The characteristics of the individual study populations are presented as means or proportions where appropriate. We evaluated the association between PPARG Pro12Ala polymorphisms and the risk of mortality in patients with CKD in each study using RR and 95% CIs. Risk ratio (RR) was used as an effect measure and the random effects model was used to combine the results. The τ2 statistic, which was estimated using the Der Simonian–Laird method, was used to assess heterogeneity, and a random-effects model was used to calculate the weighed effect size.

The I^2^ was calculated with Cochrane Q test and used to quantify study heterogeneity. An I^2^ > 50% was indicative of moderate-to-high heterogeneity (Higgins et al., 2003). Egger’s regression and funnel plots were used to evaluate the symmetry after combination results. Three common genetic models, allele type, dominant, and recessive were used to calculate the association between genetic polymorphism and the risk of mortality in patients with CKD.

Statistical analyses were conducted using the “metafor” (Viechtbauer, 2010) and “meta” (Schwarzer et al., 2015) packages of R software, version 3.3.1. We performed a Trial Sequential Analysis (TSA) to calculate the required sample size to validate whether the results of the meta-analysis present a definite conclusion (Thorlund et al., 2011). Zero event handing was set at 1. The confidence interval was set at 95%. The degrees of freedom were set at 2. The number of patients with the G allele was inputted into the Intervention group, the number of patients with the C allele was inputted into the Control group, the number of deaths was inputted into Event, and the sum of deaths and surviving patients was inputted into Total. Type 1 error was set at 0.05. Power was set at 0.95 for the estimation of sample number. The diversity value of 80% that was internally set in TSA was inputted as heterogeneity.

A RR value of 1.5 is a reasonable value for the correlation between genes and disease (Wacholder et al., 2004). However, as the G allele may be an associate factor, this study set the RR value as the reciprocal of 1.5 (i.e., 0.67). As the mortality rate of patients with the C allele was 33.1% in the study by Szeto et al. (Szeto et al., 2008) and 31.4% in the study by Chao et al. (Chao et al., 2015), this study used 30% as the mortality rate for patients with the C allele.

## 3 Results

### 3.1 Retrospective Cohort Study

The characteristics and blood biochemical values of subjects are shown in Table 1. This study retrospectively analyzed 767 hemodialysis patients, 49.9% were males (*n* = 383), the mean age was 72.19 ± 12.69 years, the degree of junior high school and below was 79.4%, the dialysis period was 7.72 ± 5.18 years, and the prevalence of diabetes and hypertension was 77.8 and 88.8%, respectively. Renal parenchymal inflammation (32.9%) was the main cause of renal disease. After the follow-up of 14.5 months, 640 patients had survived and 127 had died.

**TABLE 1:** General information of the followed-up population.

Follow-up status independent variables	All patients (n = 767)	Surviving patients (n = 640)	Deceased (n = 127)
Dialysis clinic (%)			
Dialysis Clinic 1	128 (16.7)	103 (16.1)	25 (19.7)
Dialysis Clinic 2	90 (11.7)	71 (11.1)	19 (15.0)
Dialysis Clinic 3	116 (15.1)	91 (14.2)	25 (19.7)
Dialysis Clinic 4	128 (16.7)	112 (17.5)	16 (12.6)
Dialysis Clinic 5	40 (5.2)	33 (5.2)	7 (5.5)
Dialysis Clinic 6	142 (18.5)	117 (18.3)	25 (19.7)
Dialysis Clinic 7	123 (16.0)	113 (17.7)	10 (7.9)
Male (%)	383 (49.9)	323 (50.5)	60 (47.2)
Age (mean ± SD), years	72.19 ± 12.69	71.45 ± 12.46	75.94 ± 13.19
Dialysis period (mean ± SD), years	7.72 ± 5.18	7.77 ± 5.23	7.49 ± 4.97
Diabetes (%)	326 (77.8)	266 (75.1)	60 (92.3)
Hypertension (%)	175 (88.8)	137 (88.4)	38 (90.5)
Primary cause of dialysis (%)			
Renal parenchymal inflammatory changes	252 (32.9)	221 (34.5)	31 (24.4)
Hypertensive nephropathy	164 (21.4)	133 (20.8)	31 (24.4)
Diabetic nephropathy	215 (28.0)	166 (25.9)	49 (38.6)
Others	136 (17.7)	120 (18.8)	16 (12.6)
Education level (%)			
Elementary school or illiterate	353 (46.5)	288 (45.6)	65 (51.2)
Junior high school	250 (32.9)	209 (33.1)	41 (32.3)
Senior high school	82 (10.8)	73 (11.6)	9 (7.1)
Junior college	55 (7.2)	46 (7.3)	9 (7.1)
University	19 (2.5)	16 (2.5)	3 (2.4)

Follow-up status independent variables	All patients (n = 767)	Surviving patients (n = 640)	Deceased (n = 127)
Blood biochemical values (mean ± SD)			
Leukocytes, mm^3^	9,960.30 ± 17,221.04	9,471.04 ± 15,618.00	12,418.14 ± 23,616.79
Erythrocytes, mm^3^	3.30 ± 0.62	3.32 ± 0.63	3.22 ± 0.57
Hemoglobin, g/dL	9.82 ± 1.46	9.83 ± 1.48	9.77 ± 1.39
Urea nitrogen before dialysis, mg/dL	73.92 ± 24.75	70.68 ± 21.89	90.65 ± 31.21
Urea nitrogen after dialysis, mg/dL	13.67 ± 7.91	13.54 ± 8.10	14.44 ± 6.67
Creatine, mg/dL	9.42 ± 2.78	9.46 ± 2.55	9.25 ± 3.72
Albumin, g/dL	3.58 ± 0.58	3.60 ± 0.57	3.47 ± 0.59
Urine protein, mg/dL	7.20 ± 5.65	7.25 ± 5.99	6.82 ± 0.67
Pre-prandial blood glucose, mg/dL	148.90 ± 75.40	147.39 ± 74.40	160.91 ± 83.21
Triglycerides, mg/dL	157.22 ± 98.82	156.53 ± 98.42	160.71 ± 101.14
Cholesterol, mg/dL	162.56 ± 44.96	162.83 ± 42.55	161.20 ± 55.73
Sodium, mEq/L	137.86 ± 4.53	138.14 ± 4.40	136.43 ± 4.88
Potassium, mEq/L	4.79 ± 1.95	4.80 ± 2.10	4.74 ± 0.90
Calcium, mg/dL	9.05 ± 0.89	9.07 ± 0.88	8.96 ± 0.90
Phosphorous, mg/dL	4.93 ± 1.67	4.95 ± 1.66	4.88 ± 1.74
Estimated glomerular filtration rate, mL/min/1.73 m^2^	5.72 ± 2.45	5.63 ± 2.24	6.23 ± 3.28

Table 2 shows the correlation between PPARG Pro12Ala gene polymorphisms in hemodialysis patients and the risk of all-cause mortality. Using the C allele as the reference, the G allele had a less risk of mortality (HR = 0.35, 95% CI: 0.13–0.93). After adjusting for age and gender, the G allele was still an associate factor (HR = 0.33, 95% CI: 0.12–0.88). Using the CC genotype as the reference, the HR for the CG genotype was 0.32 (95% CI: 0.12–0.88) after controlling for age and gender.

**TABLE 2:** Hazard ratio for the association between PPARG Pro12Ala polymorphism in hemodialysis patients and all-cause mortality.

Genotype	Number of deaths/Total number of patients	Number of deaths/Total number of person years	Crude-HR (95%CI)	*p* Value	Adj-HR (95%CI)^a^	*p* Value
Allele						
C Allele	250/1,471	254/1,617	1.00		1.00	
G Allele	4/63	4/74	0.35 (0.13–0.93)	0.036^b^	0.33 (0.12–0.88)	0.027^b^
Co-dominant						
CC	123/705	123/772	1.00		1.00	
CG	4/61	4/72	0.35 (0.13–0.94)	0.038^b^	0.32 (0.12–0.88)	0.026^b^
GG	0/1	0/1	0.00 (0.00–Inf)	0.993	0.00 (0.00–Inf)	0.994
Dominant Model						
CC	123/705	123/772	1.00		1.00	
CG and GG	4/62	4/73	0.34 (0.13–0.93)	0.035^b^	0.32 (0.12–0.86)	0.025^b^
Recessive Model						
CC and CG	127/766	127/844	1.00		1.00	
GG	0/1	0/1	0.00 (0.00–Inf)	0.993	0.00 (0.00–Inf)	0.994

a adjusted for age and gender.

b *p* < 0.05.

In order to achieve the best evidence hierarchies, our retrospective cohort study was added to the TSA to validate the results of the meta-analysis and evaluate where a definite conclusion could be attained.

#### 3.1.1 Meta-Analysis

**FIGURE 1:**
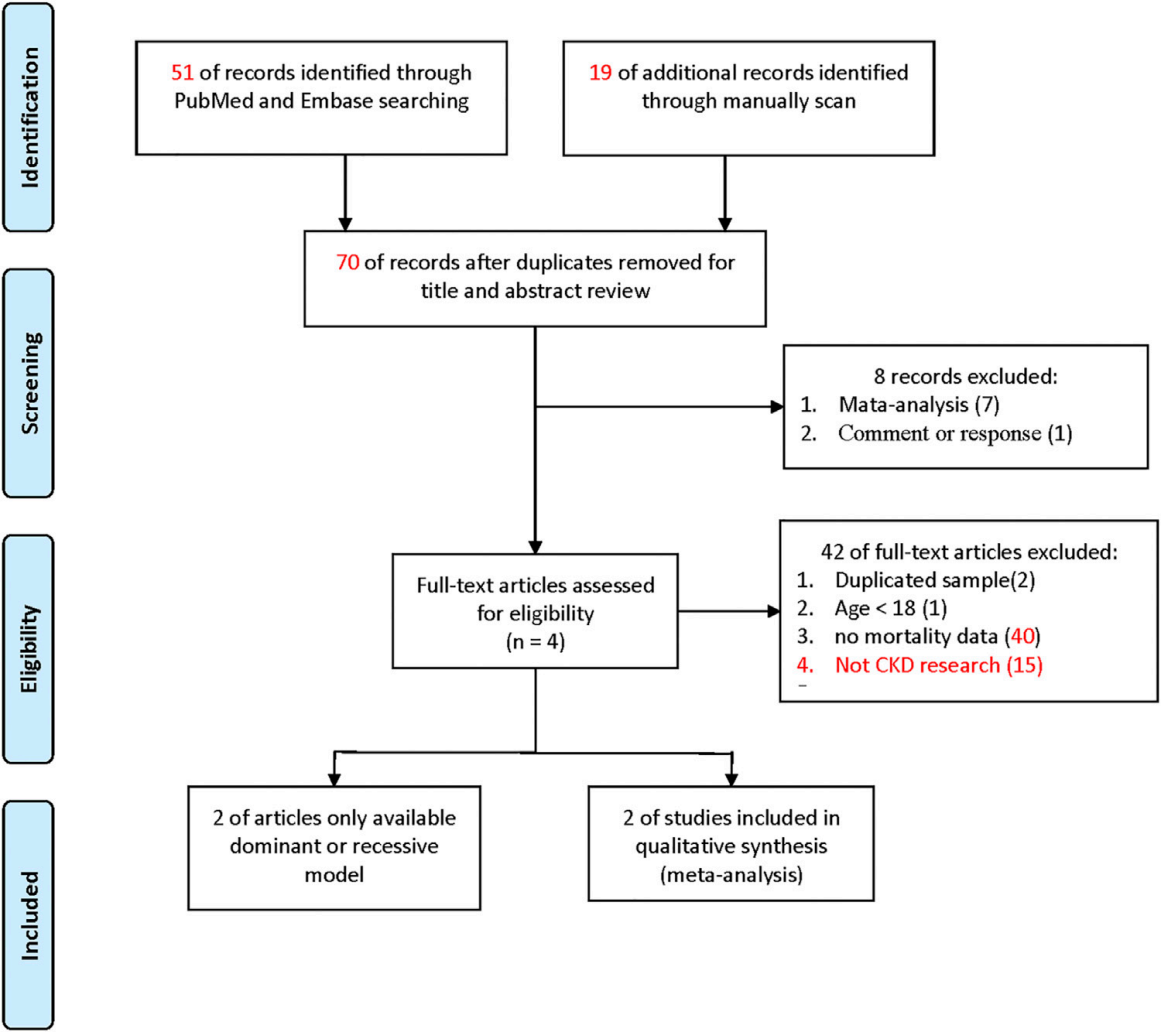
Flow diagram of the identification process for eligible studies.

Figure 1 shows the search process for eligible studies. In the present meta-analysis, we found a total of 44 articles from the PubMed and Embase databases. We also found 10 further articles from a previous meta-analysis of case-control study for investigating the association between PPARG Pro12Ala and CKD, which potentially including dialysis patients, giving 54 articles in total. Seven meta-analyses and one review article were excluded according to the basis of the title and abstract. Two articles with repeated samples on the basis of manuscript text, one article reporting a study population age of younger than 18 years, and 39 articles with no deaths were excluded. Finally, two articles were included in the meta-analysis (details are shown in Supplementary Tables S3, S4). They provided complete genotypes (C/C, C/G, and G/G genotypes).

**FIGURE 2:**
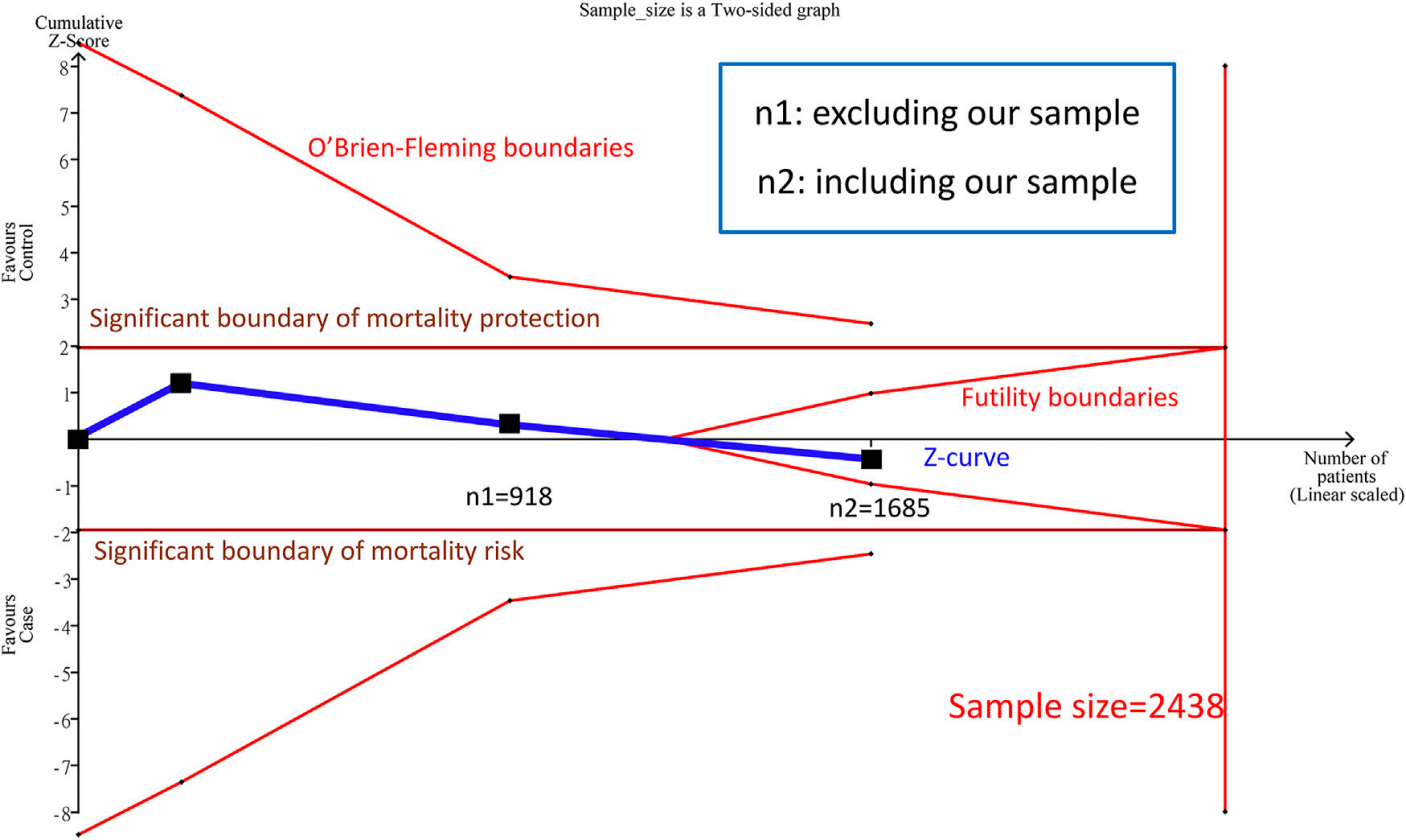
Trial Sequential Analysis (TSA) in this meta-analysis among Asians TSA is a methodology that includes a sample size calculation for a meta-analysis with the threshold of statistical significance. We performed a TSA using an allele model assumption but replaced the allele count with the sample size (divided by 2). Detailed settings: Significance level = 0.05; power = 0.95; ratio of minor to major = 1; hypothetical outcome incidence of D allele in control = 0.03; least extreme RR to be detected = 0.67; I^2^ (heterogeneity) = 8

We combined the above two study samples (n = 918) of complete genotypes by the allele model and the results were not significant (RR = 1.12, 95% CI: 0.53–2.37). The heterogeneity (I^2^) was 78.5%, and no significance was discovered between the papers (Forest plot and funnel plot shown Supplementary Figure S1). As explained in the introduction, the cumulative sample size was not big enough by TSA estimation (Figure 2) to reach a conclusive result. Therefore, we added our cohort study (n = 767) into the meta-analysis.

#### 3.1.2 TSA Conclusion From the meta-Analysis After Combining Our Cohort Study

Before combining our cohort study, TSA estimation presented that the cumulative sample size for Asian was 918 (Figure 2), and there was no significant correlation between PPARG Pro12Ala and mortality by the allele model. Nevertheless, the cumulative sample size was below the targeted sample size (*n* = 2,438); hence, no definite conclusion could be drawn from the meta-analysis for the Asian sample. Because the cumulative sample size for the Asian sample appeared to be approaching the futility area, we estimated the sample size of Asians after adding our cohort study (*n* = 767) (Figure 2).

A total of three samples (*n* = 1,685) were combined in the allele model, and the results were not significant (RR: 0.85, 95% CI: 0.39–1.83, I^2^ = 79.3%, Supplementary Figure S2). In addition, there was no potential publication bias in the paper. We found no significant correlation between PPARG Pro12Ala and mortality in the cumulative sample size for Asians was 1,685 patients, and the cumulative sample size (Z curve) entered into the futility area. Therefore, the sample size is sufficient to achieve certain results. We can prove that there was no significant correlation between PPARG Pro12Ala and mortality (allele model) in Asians, and the samples are determinant to confirm the conclusion in this study.

## 4 Discussion

The results of this cohort study showed that PPARG Pro12Ala G allele was an associate factor for risk of mortality. Similar conclusions have been reported in previous studies by Yao et al. (Yao et al., 2005) and Chao et al. (Chao et al., 2015). Previous studies also pointed out that PPARG can inhibit the inflammatory response and the proliferation of smooth muscle cells to protect against atherosclerosis (Law et al., 2000). Similar results were also observed in a previous study that reported that the G allele in PPARG Pro12Ala is an associate factor for cardiovascular diseases (Doney et al., 2004). However, Szeto et al. found that the C/G genotype is a risk factor for mortality (*p* = 0.0005) (Szeto et al., 2008). A possible reason for these contrasting findings is that the sample size is smaller and the frequency of the G allele was 2.5%, which was lower than other studies on Asians. The MAF in our study and the study by Chao et al. was 4.1 and 4.9%, respectively (Chao et al., 2015).

Jorsal et al. found that the PPARG Pro12Ala G/G genotype is a risk factor for all-cause mortality risk, with HR: 2.44 (95% CI: 1.23–4.84) among Caucasians (Jorsal et al., 2008). Although the frequency of the PPARG Pro12Ala allele is 4% in Asians, it is 11% in Caucasians. Besides the possible differences in ethnicity, the enrolled patients had Type 1 diabetes. In patients with Type 1 diabetes mellitus, the pancreas is unable to secrete insulin, leading to the inability to convert glucose into energy. However, the main cause of diabetic nephropathy in this study was Type 2 diabetes. Insulin resistance is the primary cause of type 2 diabetes mellitus and the presence of the G allele in PPARG Pro12Ala improves insulin sensitivity (Deeb et al., 1998). Therefore, it could be inferred that the G allele is an associate factor for risk of death or cardiovascular disease caused by type 2 diabetes mellitus in patients with nephropathy, but possibly not for type 1.

To the best of our knowledge, this study is the first meta-analysis in the existing literature to analyze the correlation between PPARG Pro12Ala gene polymorphisms and the risk of mortality in patients with CKD. We found that there was no association between the PPARG Pro12Ala gene polymorphisms and risk of mortality. The predictive target sample size was 2,438 using TSA and combining two previous study samples (*n* = 918). After adding 767 patients from our cohort study into the meta-analysis (cumulative sample = 1,685), under the parameter setting with the RR value of 1.5, trial sequential analysis confirmed the evidence was sufficient and conclusive. It was proven that there was no correlation between Pro12Ala gene polymorphisms in Asian patients with CKD and the risk of mortality.

The heterogeneity may be due to environmental factors (such as gender, BMI, ligand effects, medication status, dietary habits, etc.), which can be described as gene–environment interactions. Previous studies found that the G/G or G/C genotypes can significantly prevent diabetic nephropathy in Caucasians, but this was not significant in Asians. On the basis of this, we can conclude that ethnicity is an important regulatory factor (Zhang et al., 2012b; Wang et al., 2013). When the proportion of CKD patients with diabetes was 100%, PPARG Pro12Ala G was an associate factor for CKD. However, when the proportion of diabetic patients decreased, there was no significant difference. Therefore, diabetes may be a modification factor (Liu et al., 2014). In Supplementary Table S5, similar results were obtained in the stratification analysis results. Although the CIs of these estimates are quite large due to the relative small sample sizes of the two allelic and genotypic groups, this is perhaps suggestive of a difference in survivorship between diabetic and non-diabetic groups with regards to the PPARG Pro12Ala polymorphism. Thus, larger sample sizes are needed to more robustly examine this potential relationships.

Another study found that males with the G/G or G/C genotypes of PPARG Pro12 had a higher BMI, so gender and BMI have significant interactions with PPARG Pro12 gene polymorphisms (*p* = 0.039) (Morini et al., 2008). In addition, a previous study found that in samples with BMI greater than 30 kg/m2, PPARG Pro12 gene polymorphisms with C/C genotype had higher glucose level and insulin resistance. Therefore, BMI was also a potential modifier (Tönjes et al., 2006). The current studies investigated the correlation between PPARG Pro12Ala in patients with CKD, and the risk of mortality was small, so we are still unable to discuss gene–environment interactions. The accumulation of sufficient samples to discuss gene–environment interactions is a future research direction.

Several potential limitations should be noted. First, we used summary data for the meta-analysis rather than individual patient data. However, previous studies have shown that the inclusion of summary data could increase the sample size and improve the level of evidence. Second, lack of the detail information, such as personal medication and diet, they could be the effects of early and aggressive intervention with diabetes and cardiovascular protective drugs, so it may lead to an underestimation of the relationship between PPARG Pro12 and mortality. Third, during enrollment, the dialysis patients were either bedridden or on wheelchairs. Therefore, their heights could not be measured. Furthermore, only few patients had their weights measured and there was no way of knowing their BMI for correction. However, after adjusting the weights of a limited number of patients, we found that the adjusted weights were consistent with overall results but not statistically significant (data not shown). Fourth, although only two papers were located in our meta-analysis, after our study samples were also considered, we found that the futility-boundaries were touched in TSA (which means that the genetic locus and death due to kidney disease could be confirmed to be unrelated). However, heterogeneity was still high and gene–environment interactions need to be discussed. Hence, we suggest that further investigations are required to clarify the cause. In addition, the setting of the TSA parameter RR value is sophisticated. As a result, we adopt a reference-based setting to represent relatively authentic observation (Wacholder et al., 2004). All of the results complies with this operational definition. Fifth, the inference of susceptibility to CKD upon only one SNP within PPARG gene might be limited, and using all SNPs on PPARG gene, such as C161T (rs3856806), would help further understand SNPs effect on CKD.

In conclusion, our study found that PPARG Pro12Ala gene polymorphisms in Asians do not show significant correlation with the mortality risk in patients with CKD, and TSA analysis confirmed that there was no correlation. In future research, we hope to further examine the sources of the heterogeneity in the literature, such as gene–environment interactions.

## Data Availability

The original contributions presented in the study are included in the article/Supplementary Material, further inquiries can be directed to the corresponding author.
