# Supplementary figures and images for: The Effect of PPARγ rs1801282 Variant on Mortality Risk Among Asians With Chronic Kidney Disease: A Cohort Study and Meta-Analysis
Source: Front Genet. 2022 Feb 21;13:705272. doi: 10.3389/fgene.2022.705272 (PMC8898960; doi:10.3389/fgene.2022.705272)

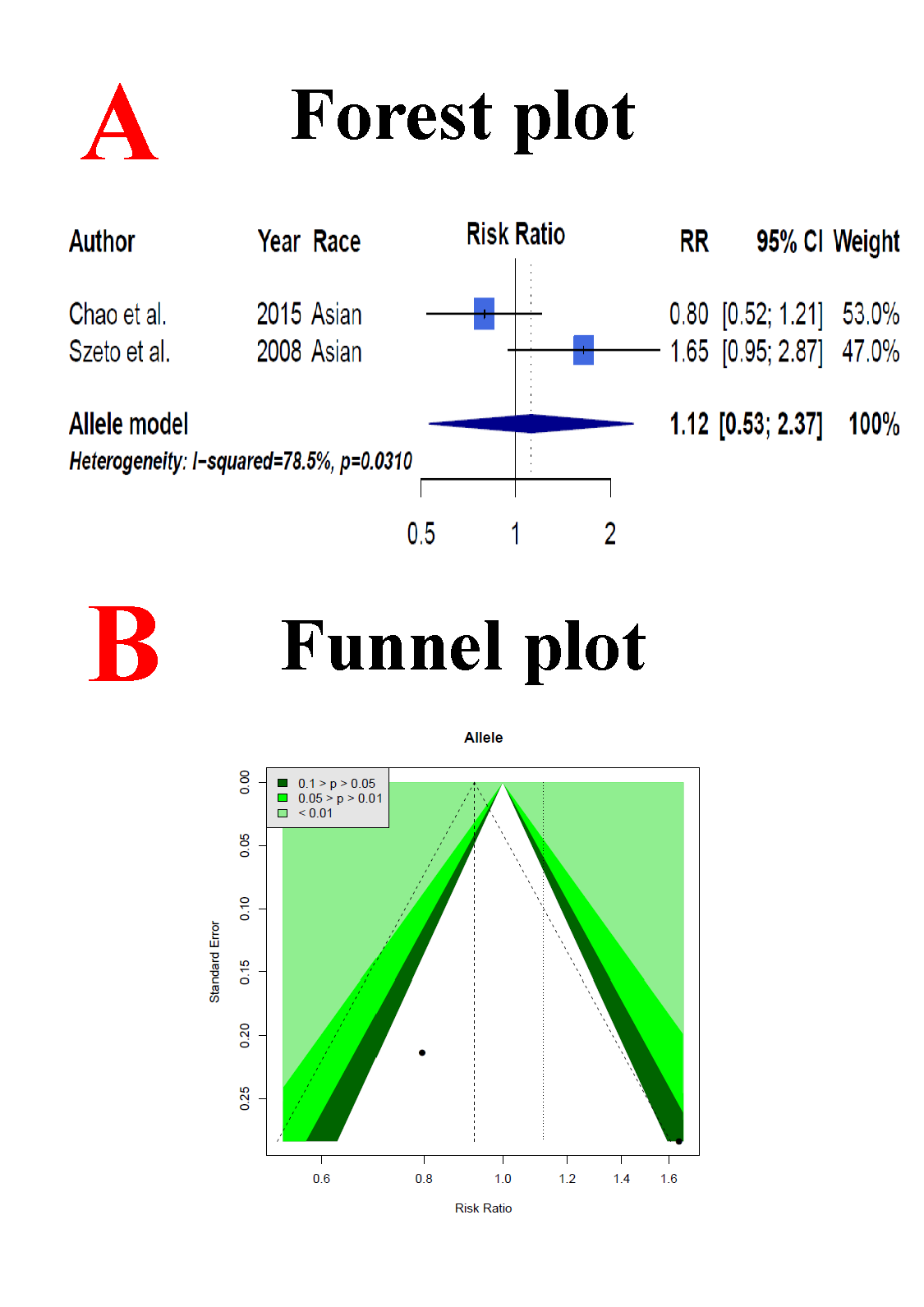

Supplement: Supplementary file 1 [file Image1.TIFF]

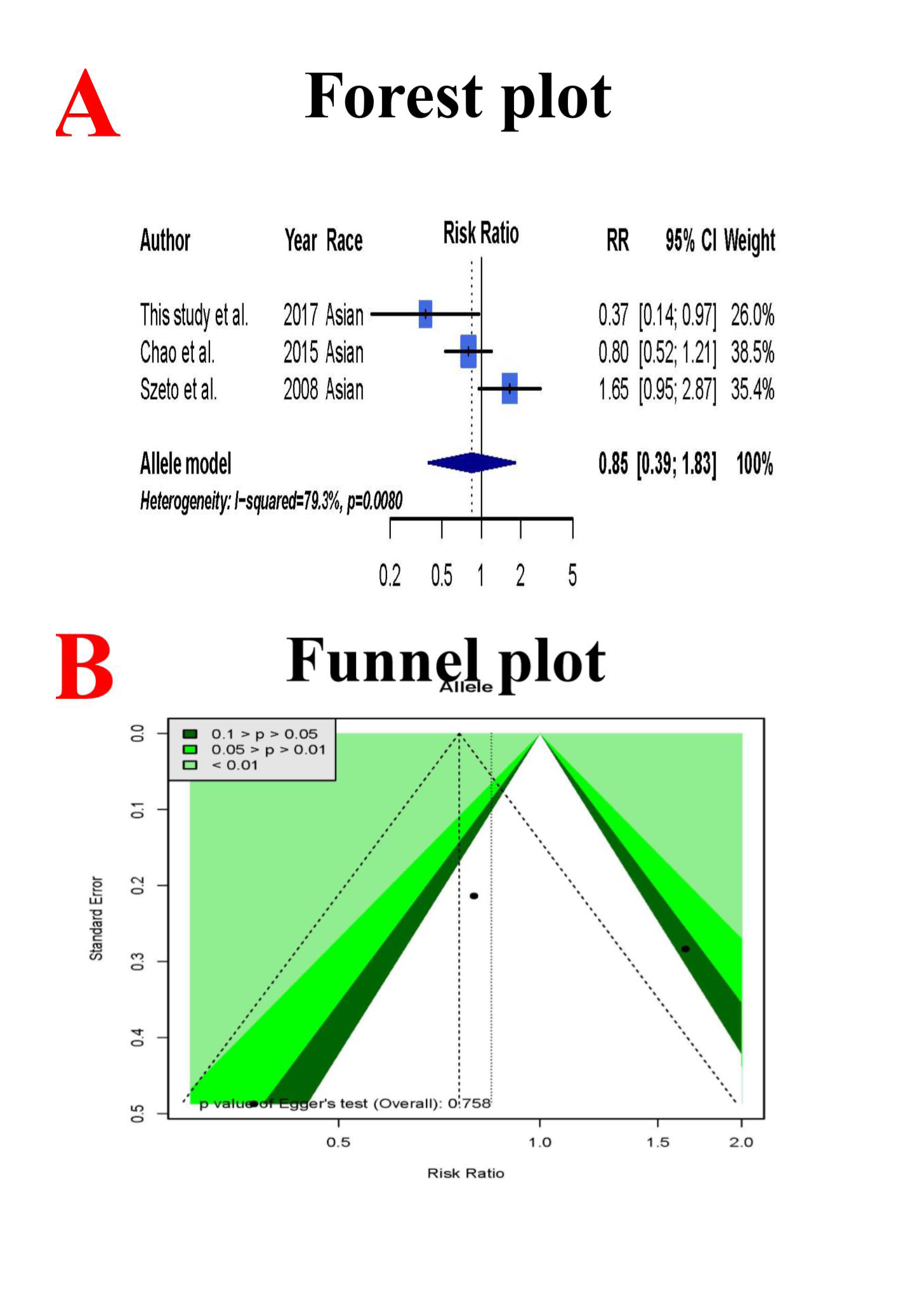

Supplement: Supplementary file 7 [file Image2.TIFF]
