# Supplementary material for: The Effect of PPARγ rs1801282 Variant on Mortality Risk Among Asians With Chronic Kidney Disease: A Cohort Study and Meta-Analysis
Source: Front Genet. 2022 Feb 21;13:705272. doi: 10.3389/fgene.2022.705272 (PMC8898960; doi:10.3389/fgene.2022.705272)
Supplement: Supplementary file 3 [file Table2.DOCX]

**Table S2. Search strategies and detailed records.**

| **Relevant text of PPAR Pro12Ala**   1. Peroxisome Proliferator-Activated Receptor gamma 2. PPAR gamma 3. mPPARgamma 4. mPPARgamma1 5. mPPARgamma2 6. PPARgamma 7. PPARgamma2 8. PPARgamma3 9. Thiazolidinedione Receptor 10. Pro12Ala 11. Pro12Ala 12. SNP 13. rs1801282 14. ((1 or 2 or 3 or 4 or 5 or 6 or 7 or 8 or 9) and ((10 or 11 or 12)) or 13   **Relevant text of chronic kidney disease**   1. Renal Insufficiency, Chronic 2. Chronic kidney disease 3. Chronic Kidney Insufficiency 4. Chronic Renal Diseases 5. Chronic Renal Insufficiency | 1. Kidney Insufficiency, Chronic 2. CKD 3. Kidney Failure, Chronic 4. Chronic Kidney Failure 5. End-Stage Kidney Disease 6. End-Stage Renal Disease 7. End-Stage Renal Failure 8. Renal Disease, End-Stage 9. Renal Failure, Chronic 10. Renal Failure, End-Stage 11. ESKD 12. ESRD 13. ESRF 14. Proteinuria 15. Albuminuria 16. Nephropathy 17. Nephritis 18. Dialysis 19. Glomerular filtration rate 20. GFR 21. Mortality 22. 15 or 16 or 17 or 18 or 19 or 20 or 21 or 22 or 23 or 24 or 25 or 26 or 27 or 28 or 29 or 30 or 31 or 32 or 33 or 34 or 35 or 36 or 37 or 38 or 39 or 40   **Combined (Final strategy)**   1. 14 and 41 |
| --- | --- |

Web sites and uniform resource locator:

**PUBMED**: <http://www.ncbi.nlm.nih.gov/pubmed>

**EMBASE**: https://www.embase.com

**Records from PUBMED** [1-36]

Mata-analysis [1-6], Comment or response [7], Duplicated sample [8, 11], Age < 18 [12], no mortality data[9, 13-21, 23-31, 33, 35, 36], Included studies[10, 22, 32, 34]

**Records from EMBASE** [37-44]

Mata-analysis [37]**,** no mortality data[38-44]

**Records through manually scan**[45-54]

**Reference**

1. Li T, Shi Y, Yin J, Qin Q, Wei S, Nie S, et al. The association between lipid metabolism gene polymorphisms and nephropathy in type 2 diabetes: a meta-analysis. Int Urol Nephrol. 2015;47(1):117-30. Epub 2014/09/30. doi: 10.1007/s11255-014-0843-6. PubMed PMID: 25262148.

2. Ding J, Zhu C, Mei X, Zhou Y, Feng B, Guo Z. Peroxisome proliferator-activated receptor gamma Pro12Ala polymorphism decrease the risk of diabetic nephropathy in type 2 diabetes: a meta analysis. Int J Clin Exp Med. 2015;8(5):7655-60. Epub 2015/07/30. PubMed PMID: 26221313; PubMed Central PMCID: PMCPMC4509258.

3. Zhou TB, Guo XF, Yin SS. Association of peroxisome proliferator-activated receptor gamma Pro12Ala gene polymorphism with type 2 diabetic nephropathy risk in Caucasian population. J Recept Signal Transduct Res. 2014;34(3):180-4. Epub 2013/12/18. doi: 10.3109/10799893.2013.868905. PubMed PMID: 24329532.

4. Liu G, Zhou TB, Jiang Z, Zheng D, Yuan F, Li Y, et al. Relationship between PPARgamma Pro12Ala gene polymorphism and type 2 diabetic nephropathy risk in Asian population: results from a meta-analysis. J Recept Signal Transduct Res. 2014;34(2):131-6. Epub 2013/12/07. doi: 10.3109/10799893.2013.864678. PubMed PMID: 24303936.

5. Wang L, Teng Z, Cai S, Wang D, Zhao X, Yu K. The association between the PPARgamma2 Pro12Ala polymorphism and nephropathy susceptibility in type 2 diabetes: a meta-analysis based on 9,176 subjects. Diagn Pathol. 2013;8:118. Epub 2013/07/17. doi: 10.1186/1746-1596-8-118. PubMed PMID: 23856170; PubMed Central PMCID: PMCPMC3751054.

6. Yu R, Bo H, Huang S. Association between the PPARG gene polymorphism and the risk of diabetic nephropathy: a meta-analysis. Genet Test Mol Biomarkers. 2012;16(5):429-34. Epub 2011/11/23. doi: 10.1089/gtmb.2011.0242. PubMed PMID: 22103651.

7. Lapice E, Cocozza S, Riccardi G, Vaccaro O. Comment on: Zhang et al. Peroxisome proliferator-activated receptor gamma polymorphism Pro12Ala is associated with nephropathy in type 2 diabetes: evidence from meta-analysis of 18 studies. Diabetes Care 2012;35:1388-1393. Diabetes Care. 2013;36(1):e18. Epub 2012/12/25. doi: 10.2337/dc12-1993. PubMed PMID: 23264301; PubMed Central PMCID: PMCPMC3526245.

8. Chao CT, Huang JW, Chiang CK, Chen YC, Fang CC, Hu FC, et al. Diabetes mellitus, superoxide dismutase and peroxisome proliferator activated receptor gamma polymorphisms modify the outcome of end-stage renal disease patients of Han Chinese origin. Nephrology (Carlton, Vic). 2016. Epub 2016/12/08. doi: 10.1111/nep.12975. PubMed PMID: 27925431.

9. Lapice E, Monticelli A, Cocozza S, Pinelli M, Cocozza S, Bruzzese D, et al. The PPARgamma2 Pro12Ala variant is protective against progression of nephropathy in people with type 2 diabetes. J Transl Med. 2015;13:85. Epub 2015/04/19. doi: 10.1186/s12967-015-0448-6. PubMed PMID: 25889595; PubMed Central PMCID: PMCPMC4358785.

10. Szeto CC, Chow KM, Poon PY, Kwan BC, Li PK. Peroxisome proliferator-activated receptor-gamma gene polymorphism and risk of cardiovascular disease in patients with diabetic nephropathy. Am J Nephrol. 2008;28(5):715-22. Epub 2008/04/18. doi: 10.1159/000127452. PubMed PMID: 18417957.

11. Chao CT, Chen YC, Chiang CK, Huang JW, Fang CC, Chang CC, et al. Interplay between Superoxide Dismutase, Glutathione Peroxidase, and Peroxisome Proliferator Activated Receptor Gamma Polymorphisms on the Risk of End-Stage Renal Disease among Han Chinese Patients. Oxid Med Cell Longev. 2016;2016:8516748. Epub 2016/02/18. doi: 10.1155/2016/8516748. PubMed PMID: 26881045; PubMed Central PMCID: PMCPMC4736813.

12. Jin J, Ding G, Bao H, Chen Y, Han Y, Zhao F, et al. Correlation between PPAR Gene Polymorphisms and Primary Nephrotic Syndrome in Children. PPAR Res. 2013;2013:927915. Epub 2013/10/11. doi: 10.1155/2013/927915. PubMed PMID: 24109487; PubMed Central PMCID: PMCPMC3786523.

13. Hosseini SM, Boright AP, Sun L, Canty AJ, Bull SB, Klein BE, et al. The association of previously reported polymorphisms for microvascular complications in a meta-analysis of diabetic retinopathy. Hum Genet. 2015;134(2):247-57. Epub 2014/12/10. doi: 10.1007/s00439-014-1517-2. PubMed PMID: 25487307; PubMed Central PMCID: PMCPMC4291513.

14. Mohamed Youssef S, Mohamed N, Afef S, Khaldoun BH, Fadoua N, Fadhel NM, et al. Interaction Effects of the Leu162Val PPAR alpha and Pro12Ala PPAR gamma 2 Gene Variants with Renal Function in Metabolic Syndrome Population. PPAR Res. 2013;2013:329862. Epub 2013/05/22. doi: 10.1155/2013/329862. PubMed PMID: 23690758; PubMed Central PMCID: PMCPMC3649708.

15. Hocher B, Schlemm L, Haumann H, Poralla C, Chen YP, Li J, et al. Interaction of maternal peroxisome proliferator-activated receptor gamma2 Pro12Ala polymorphism with fetal sex affects maternal glycemic control during pregnancy. Pharmacogenet Genomics. 2010;20(2):139-42. Epub 2009/12/25. doi: 10.1097/FPC.0b013e3283357337. PubMed PMID: 20032817.

16. Malecki MT, Cyganek K, Mirkiewicz-Sieradzka B, Wolkow PP, Wanic K, Skupien J, et al. Alanine variant of the Pro12Ala polymorphism of the PPARgamma gene might be associated with decreased risk of diabetic retinopathy in type 2 diabetes. Diabetes research and clinical practice. 2008;80(1):139-45. Epub 2007/12/14. doi: 10.1016/j.diabres.2007.11.001. PubMed PMID: 18077048.

17. Smith WM, Zhou XP, Kurose K, Gao X, Latif F, Kroll T, et al. Opposite association of two PPARG variants with cancer: overrepresentation of H449H in endometrial carcinoma cases and underrepresentation of P12A in renal cell carcinoma cases. Hum Genet. 2001;109(2):146-51. Epub 2001/08/21. PubMed PMID: 11511919.

18. Gayathri SB, Radha V, Vimaleswaran KS, Mohan V. Association of the PPARGC1A gene polymorphism with diabetic nephropathy in an Asian Indian population (CURES-41). Metab Syndr Relat Disord. 2010;8(2):119-26. Epub 2009/11/11. doi: 10.1089/met.2009.0040. PubMed PMID: 19900151.

19. Omori S, Tanaka Y, Takahashi A, Hirose H, Kashiwagi A, Kaku K, et al. Association of CDKAL1, IGF2BP2, CDKN2A/B, HHEX, SLC30A8, and KCNJ11 with susceptibility to type 2 diabetes in a Japanese population. Diabetes. 2008;57(3):791-5. Epub 2007/12/29. doi: 10.2337/db07-0979. PubMed PMID: 18162508.

20. Sale MM, Smith SG, Mychaleckyj JC, Keene KL, Langefeld CD, Leak TS, et al. Variants of the transcription factor 7-like 2 (TCF7L2) gene are associated with type 2 diabetes in an African-American population enriched for nephropathy. Diabetes. 2007;56(10):2638-42. Epub 2007/07/03. doi: 10.2337/db07-0012. PubMed PMID: 17601994.

21. Zambrano-Galván G, Reyes-Romero MA, Lazalde B, Rodríguez-Morán M, Guerrero-Romero F. Risk of microalbuminuria in relatives of subjects with diabetic nephropathy: A predictive model based on multivariable dimensionality reduction approach. Clinical Nephrology. 2015;83(2):86-92.

22. Chao CT, Chen YC, Chiang CK, Huang JW, Hu FC, Fang CC, et al. Sequence variants of peroxisome proliferator-activated receptor-gamma gene and the clinical courses of patients with end-stage renal disease. Dis Markers. 2015;2015:763459. Epub 2015/03/19. doi: 10.1155/2015/763459. PubMed PMID: 25784779; PubMed Central PMCID: PMCPMC4345048.

23. Yang B, Zhao H, Millward BA, Demaine AG. The Rate of Decline of Glomerular Filtration Rate May Not Be Associated with Polymorphism of the PPAR2 Gene in Patients with Type 1 Diabetes and Nephropathy. PPAR Res. 2014;2014:523584. Epub 2014/03/04. doi: 10.1155/2014/523584. PubMed PMID: 24587794; PubMed Central PMCID: PMCPMC3920619.

24. Liu F, Mei X, Zhang Y, Qi H, Wang J, Wang Y, et al. Association of peroxisome proliferator-activated receptorγ gene Pro12Ala and C161T polymorphisms with cardiovascular risk factors in maintenance hemodialysis patients. Molecular Biology Reports. 2014;41(11):7555-65.

25. Azab MM, Abdel-Azeez HA, Zanaty MF, El Alawi SM. Peroxisome proliferator activated receptor gamma2 gene Pro12Ala gene polymorphism in type 2 diabetes and its relationship with diabetic nephropathy. Clin Lab. 2014;60(5):743-9. Epub 2014/05/21. PubMed PMID: 24839816.

26. Bhaskar LV, Mahin S, Ginila RT, Soundararajan P. Role of the ACE ID and PPARG P12A Polymorphisms in Genetic Susceptibility of Diabetic Nephropathy in a South Indian Population. Nephrourol Mon. 2013;5(3):813-7. Epub 2013/11/28. doi: 10.5812/numonthly.9573. PubMed PMID: 24282791; PubMed Central PMCID: PMCPMC3830907.

27. Zhang H, Zhu S, Chen J, Tang Y, Hu H, Mohan V, et al. Peroxisome proliferator-activated receptor gamma polymorphism Pro12Ala Is associated with nephropathy in type 2 diabetes: evidence from meta-analysis of 18 studies. Diabetes Care. 2012;35(6):1388-93. Epub 2012/05/24. doi: 10.2337/dc11-2142. PubMed PMID: 22619290; PubMed Central PMCID: PMCPMC3357218.

28. De Cosmo S, Prudente S, Lamacchia O, Lapice E, Morini E, Di Paola R, et al. PPARgamma2 P12A polymorphism and albuminuria in patients with type 2 diabetes: a meta-analysis of case-control studies. Nephrology, dialysis, transplantation : official publication of the European Dialysis and Transplant Association - European Renal Association. 2011;26(12):4011-6. Epub 2011/04/16. doi: 10.1093/ndt/gfr187. PubMed PMID: 21493814.

29. Liu L, Zheng T, Wang F, Wang N, Song Y, Li M, et al. Pro12Ala polymorphism in the PPARG gene contributes to the development of diabetic nephropathy in Chinese type 2 diabetic patients. Diabetes Care. 2010;33(1):144-9. Epub 2009/10/20. doi: 10.2337/dc09-1258. PubMed PMID: 19837787; PubMed Central PMCID: PMCPMC2797960.

30. Lapice E, Pinelli M, Riccardi G, Vaccaro O. Pro12Ala polymorphism in the PPARG gene contributes to the development of diabetic nephropathy in Chinese type 2 diabetic patients: comment on the study by Liu et al. Diabetes Care. 2010;33(8):e114; author reply e5. Epub 2010/07/30. doi: 10.2337/dc10-0596. PubMed PMID: 20668149.

31. De Cosmo S, Motterlini N, Prudente S, Pellegrini F, Trevisan R, Bossi A, et al. Impact of the PPAR-gamma2 Pro12Ala polymorphism and ACE inhibitor therapy on new-onset microalbuminuria in type 2 diabetes: evidence from BENEDICT. Diabetes. 2009;58(12):2920-9. Epub 2009/09/02. doi: 10.2337/db09-0407. PubMed PMID: 19720797; PubMed Central PMCID: PMCPMC2780880.

32. Jorsal A, Tarnow L, Lajer M, Ek J, Hansen T, Pedersen O, et al. The PPAR gamma 2 Pro12Ala variant predicts ESRD and mortality in patients with type 1 diabetes and diabetic nephropathy. Mol Genet Metab. 2008;94(3):347-51. Epub 2008/05/10. doi: 10.1016/j.ymgme.2008.03.014. PubMed PMID: 18467141.

33. Pollex RL, Mamakeesick M, Zinman B, Harris SB, Hegele RA, Hanley AJ. Peroxisome proliferator-activated receptor gamma polymorphism Pro12Ala is associated with nephropathy in type 2 diabetes. J Diabetes Complications. 2007;21(3):166-71. Epub 2007/05/12. doi: 10.1016/j.jdiacomp.2006.02.006. PubMed PMID: 17493550.

34. Yao Q, Nordfors L, Axelsson J, Heimburger O, Qureshi AR, Barany P, et al. Peroxisome proliferator-activated receptor gamma polymorphisms affect systemic inflammation and survival in end-stage renal disease patients starting renal replacement therapy. Atherosclerosis. 2005;182(1):105-11. Epub 2005/08/24. doi: 10.1016/j.atherosclerosis.2005.01.033. PubMed PMID: 16115480.

35. Caramori ML, Canani LH, Costa LA, Gross JL. The human peroxisome proliferator-activated receptor gamma2 (PPARgamma2) Pro12Ala polymorphism is associated with decreased risk of diabetic nephropathy in patients with type 2 diabetes. Diabetes. 2003;52(12):3010-3. Epub 2003/11/25. PubMed PMID: 14633865.

36. Herrmann SM, Ringel J, Wang JG, Staessen JA, Brand E. Peroxisome proliferator-activated receptor-gamma2 polymorphism Pro12Ala is associated with nephropathy in type 2 diabetes: The Berlin Diabetes Mellitus (BeDiaM) Study. Diabetes. 2002;51(8):2653-7. Epub 2002/07/30. PubMed PMID: 12145184.

37. Cao Y, Zhao H, Yan M, Zhang B, Li P. Meta-analysis of the association of Prol2Ala polymorphism of peroxisome proliferator activated receptor gamma gene with diabetic kidney disease in Chinese Han population. Hong Kong Journal of Nephrology. 2015;17(2):S8.

38. Algenabi AHAF, Hadi BaA. Study of PPARG2 Gene Polymorphism (Pro12Ala) in Iraqi Patients with Type 2 Diabetes Mellitus. 2016.

39. Liu YQ, Wang LC, Dong J, Wang JF, Wu SJ. Association of Pro12Ala polymorphism of PPAR-γ2 Gene and diabetic retinopathy in type 2 diabetes. International Eye Science. 2013;13(6):1108-11.

40. Kuo TY, Kang MJ, Chen JW, Ho HY, Ting CT, Lin TH, et al. A two-stage matched case-control study on multiple hypertensive candidate genes in han Chinese. American Journal of Hypertension. 2012;25(7):804-11.

41. Wang SY, Tang SS, Hu C, Jia WP. The association between FNDC5 gene polymorphisms and diabetic retinopathy in Chinese type 2 diabetes patients. Diabetes research and clinical practice. 2014;106:S89.

42. Kurzawski M, Dziewanowski K, Łapczuk J, Wajda A, Droździk M. Analysis of common type 2 diabetes mellitus genetic risk factors in new-onset diabetes after transplantation in kidney transplant patients medicated with tacrolimus. European Journal of Clinical Pharmacology. 2012;68(12):1587-94.

43. Avzaletdinova DS, Sharipova LF, Kochetova OV, Morugova TV, Erdman VV, Mustafina OE. Association of variable rs1801282 locus of PPARG2 gene with diabetic nephropathy. Russian Journal of Genetics. 2016;52(8):877-81.

44. Osman NA, NasrAllah MM, Kamal MM, Ahmed AI. The association between diabetic nephropathy and polymorphisms of PPAR PRO12ALA and CCR5 32 genes in type 2 diabetes. Nephrology Dialysis Transplantation. 2013;28:i382-i3.

45. Herrmann S-M, Ringel J, Wang J-G, Staessen JA, Brand E. Peroxisome proliferator-activated receptor-γ2 polymorphism Pro12Ala is associated with nephropathy in type 2 diabetes: the Berlin Diabetes Mellitus (BeDiaM) Study. Diabetes. 2002;51(8):2653-7.

46. Pollex RL, Mamakeesick M, Zinman B, Harris SB, Hegele RA, Hanley AJ. Peroxisome proliferator-activated receptor γ polymorphism Pro12Ala is associated with nephropathy in type 2 diabetes. Journal of diabetes and its complications. 2007;21(3):166-71.

47. Mori H, Ikegami H, Kawaguchi Y, Seino S, Yokoi N, Takeda J, et al. The Pro12→ Ala substitution in PPAR-γ is associated with resistance to development of diabetes in the general population: possible involvement in impairment of insulin secretion in individuals with type 2 diabetes. Diabetes. 2001;50(4):891-4.

48. Caramori ML, Canani LH, Costa LA, Gross JL. The human peroxisome proliferator-activated receptor γ2 (PPARγ2) Pro12Ala polymorphism is associated with decreased risk of diabetic nephropathy in patients with type 2 diabetes. Diabetes. 2003;52(12):3010-3.

49. De Cosmo S, Motterlini N, Prudente S, Pellegrini F, Trevisan R, Bossi A, et al. Impact of the PPAR-γ2 Pro12Ala polymorphism and ACE inhibitor therapy on new-onset microalbuminuria in type 2 diabetes: evidence from BENEDICT. Diabetes. 2009;58(12):2920-9.

50. Lapice E, Pinelli M, Riccardi G, Vaccaro O. Pro12Ala polymorphism in the PPARG gene contributes to the development of diabetic nephropathy in Chinese type 2 diabetic patients: comment on the study by Liu et al. Diabetes Care. 2010;33(8):e114-e.

51. Deeb SS, Fajas L, Nemoto M, Pihlajamäki J, Mykkänen L, Kuusisto J, et al. A Pro12Ala substitution in PPARγ2 associated with decreased receptor activity, lower body mass index and improved insulin sensitivity. Nature genetics. 1998;20(3):284.

52. Maeda A, Gohda T, Funabiki K, Horikoshi S, Tomino Y. Peroxisome proliferator‐activated receptor γ gene polymorphism is associated with serum triglyceride levels and body mass index in Japanese type 2 diabetic patients. Journal of clinical laboratory analysis. 2004;18(6):317-21.

53.　Erdogan M, Karadeniz M, Eroglu Z, Tezcanl B, Selvi N, Yilmaz C. The relationship of the peroxisome proliferator-activated receptor-gamma 2 exon 2 and exon 6 gene polymorphism in Turkish type 2 diabetic patients with and without nephropathy. Diabetes Res Clin Pract. 2007; 78(3): 355-9.

54.　Liu L, Zheng T, Wang F, Wang N, Song Y, Li M et al. Pro12Ala polymorphism in the PPARG gene contributes to the development of diabetic nephropathy in Chinese type 2 diabetic patients. Diabetes Care. 2010; 33(1):144–9.
