# Supplementary material for: The Effect of PPARγ rs1801282 Variant on Mortality Risk Among Asians With Chronic Kidney Disease: A Cohort Study and Meta-Analysis
Source: Front Genet. 2022 Feb 21;13:705272. doi: 10.3389/fgene.2022.705272 (PMC8898960; doi:10.3389/fgene.2022.705272)
Supplement: Supplementary file 4 [file Table5.DOCX]

**Table S5**. Hazard ratio for the association between PPARG Pro12Ala polymorphism in hemodialysis patients with diabetes or not and all-cause mortality

| **Genotype** | **Number of deaths/Total number of patients** | **Crude-HR (95% CI)** | **P value** | **Adj-HR (95% CI)^#^** | **P value** |
| --- | --- | --- | --- | --- | --- |
| Diabetic |  |  |  |  |  |
| Allele |  |  |  |  |  |
| C Allele | 119/628 | 1 |  | 1 |  |
| G Allele | 1/24 | 0.20 (0.03 - 1.42) | 0.107 | 0.16 (0.02 - 1.15) | 0.069 |
| Co-dominant |  |  |  |  |  |
| CC | 59/302 | 1 |  | 1 |  |
| CG | 1/24 | 0.19 (0.03 - 1.38) | 0.101 | 0.15 (0.02 - 1.11) | 0.063 |
| GG | 0/0 | NA |  | NA |  |
| Dominant Model |  |  |  |  |  |
| CC | 59/302 | 1 |  | 1 |  |
| CG and GG | 1/24 | 0.19 (0.03 - 1.38) | 0.101 | 0.15 (0.02 - 1.11) | 0.063 |
| Recessive Model |  |  |  |  |  |
| CC and CG | 60/326 | 1 |  | 1 |  |
| GG | 0/0 | NA |  | NA |  |
| Non-diabetic |  |  |  |  |  |
| Allele |  |  |  |  |  |
| C Allele | 131/843 | 1 |  | 1 |  |
| G Allele | 3/39 | 0.47 (0.15 - 1.47) | 0.193 | 0.47 (0.15 - 1.49) | 0.203 |
| Co-dominant |  |  |  |  |  |
| CC | 64/403 | 1 |  | 1 |  |
| CG | 3/37 | 0.48 (0.15 - 1.53) | 0.216 | 0.49 (0.15 - 1.56) | 0.226 |
| GG | 0/1 | 0.00 (0.00 - Inf) | 0.996 | 0.00 (0.00 - Inf) | 0.996 |
| Dominant Model |  |  |  |  |  |
| CC | 64/403 | 1 |  | 1 |  |
| CG and GG | 3/38 | 0.47 (0.15 - 1.49) | 0.200 | 0.48 (0.15 - 1.52) | 0.209 |
| Recessive Model |  |  |  |  |  |
| CC and CG | 67/440 | 1 |  | 1 |  |
| GG | 0/1 | 0.00 (0.00 - Inf) | 0.996 | 0.00 (0.00 - Inf) | 0.996 |

#adjusted for age and gender

*p < 0.05
