# Supplementary material for: The Effect of PPARγ rs1801282 Variant on Mortality Risk Among Asians With Chronic Kidney Disease: A Cohort Study and Meta-Analysis
Source: Front Genet. 2022 Feb 21;13:705272. doi: 10.3389/fgene.2022.705272 (PMC8898960; doi:10.3389/fgene.2022.705272)
Supplement: Supplementary file 5 [file Table3.DOCX]

**Table S3.** General description of articles included in the meta-analysis

| **Author** | **Year** | **Sex** ^a^ | **BMI** ^b^ | **DN** ^c^ | **Dead group** | | | | **Alive group** | | |
| --- | --- | --- | --- | --- | --- | --- | --- | --- | --- | --- | --- |
|  |  |  |  |  | **CC** | **CG** | | **GG** | **CC** | **CG** | **GG** |
| This Study | 2017 | 47.3 |  | 38.8 | 123 | 4 | | 0 | 582 | 57 | 1 |
| Chao | 2015 | 47.9 |  | 33.8 | 200 | 17 | 0 | | 435 | 41 | 5 |
| Szeto | 2008 | 41.4 | 22.1 | 100 | 68 | 6 | | 0 | 141 | 5 | 0 |

^a^: Male ratio, percentage; ^b^: Body Mass Index, kg/m^2^; ^c^: Prevalence of Diabetes mellitus, percentage
