# Supplementary material for: The Effect of PPARγ rs1801282 Variant on Mortality Risk Among Asians With Chronic Kidney Disease: A Cohort Study and Meta-Analysis
Source: Front Genet. 2022 Feb 21;13:705272. doi: 10.3389/fgene.2022.705272 (PMC8898960; doi:10.3389/fgene.2022.705272)
Supplement: Supplementary file 6 [file Table4.DOCX]

| **Author** | **Year** | **Country** | **Ethnicity** | **Study**  **design^a^** | **CKD type^b^** | **Kidney function**  **of case** ^c^ | **Definition of case group** ^d^ |
| --- | --- | --- | --- | --- | --- | --- | --- |
| This Study | 2017 | Taiwan | Asian | CS | Mixed | ESRD | HD |
| Chao | 2015 | Taiwan | Asian | CS | Mixed | ESRD | RRT |
| Szeto | 2008 | China | Asian | CS | T2DN | non-ESRD | creatinine > 150 mol/l or CCr <60 ml/min |

**Table S4.** Information extracted from articles that was included in the meta-analysis

**^a^**:CS –Cohort Study.

^b^: T1DN – Type 1 diabetic nephropathy; T2DN –Type 2 diabetic nephropathy; Mixed –Diabetic nephropathy, hypertensive nephropathy or kidney inflammation

^c^: ESRD – only ESRD patients; non-ESRD – not only ESRD patients.

^d^:HD – hemodialysis; CCr – creatinine clearance; RRT – renal replacement therapy
